# An External Validation Study on Two Pre-Trained Large Language Models for Multimodal Prognostication in Laryngeal and Hypopharyngeal Cancer: Integrating Clinical, Treatment, and Radiomic Data to Predict Survival Outcomes with Interpretable Reasoning

**DOI:** 10.3390/bioengineering12121345

**Published:** 2025-12-10

**Authors:** Wing-Keen Yap, Shih-Chun Cheng, Chia-Hsin Lin, Ing-Tsung Hsiao, Tsung-You Tsai, Wing-Lake Yap, Willy Po-Yuan Chen, Chien-Yu Lin, Shih-Ming Huang

**Affiliations:** 1 Department of Radiation Oncology, Proton and Radiation Therapy Center, Linkou Chang Gung Memorial Hospital, College of Medicine, Chang Gung University, Kwei-Shan, Taoyuan 333, Taiwan; carsonyap2010@gmail.com (W.-K.Y.); alex19901016@hotmail.com (C.-H.L.); willy84213@cgmh.org.tw (W.P.-Y.C.); qqvirus1022@gmail.com (C.-Y.L.); 2 Department of Medical Imaging and Radiological Sciences, College of Medicine, Chang Gung University, Taoyuan 333, Taiwan; andys0975@gmail.com; 3 UTHealth Graduate School of Biomedical Sciences, The University of Texas MD Anderson Cancer Center, Houston, TX 77030, USA; 4 Department of Medical Imaging and Radiological Sciences, Healthy Aging Research Center, Chang Gung University, Taoyuan 333, Taiwan; ihsiao@mail.cgu.edu.tw; 5 Department of Otolaryngology—Head and Neck Surgery, Linkou Chang Gung Memorial Hospital, College of Medicine, Chang Gung University, Kwei-Shan, Taoyuan 333, Taiwan; j-acky_101@hotmail.com; 6 Department of Post-Baccalaureate Medicine, Kaohsiung Medical University, Kaohsiung 807, Taiwan; alex_yap2008@hotmail.com; 7 Department of Radiation Oncology, Keelung Chang Gung Memorial Hospital, Keelung 204, Taiwan

**Keywords:** large language models, laryngeal cancer, hypopharyngeal cancer, radiomics, prognosis, survival prediction

## Abstract

**Background:** Laryngeal and hypopharyngeal cancers (LHCs) exhibit heterogeneous outcomes after definitive radiotherapy (RT). Large language models (LLMs) may enhance prognostic stratification by integrating complex clinical and imaging data. This study validated two pre-trained LLMs—GPT-4o-2024-08-06 and Gemma-2-27b-it—for outcome prediction in LHC. **Methods:** Ninety-two patients with non-metastatic LHC treated with definitive (chemo)radiotherapy at Linkou Chang Gung Memorial Hospital (2006–2013) were retrospectively analyzed. First-order and 3D radiomic features were extracted from intra- and peritumoral regions on pre- and mid-RT CT scans. LLMs were prompted with clinical variables, radiotherapy notes, and radiomic features to classify patients as high- or low-risk for death, recurrence, and distant metastasis. Model performance was assessed using sensitivity, specificity, AUC, Kaplan–Meier survival analysis, and McNemar tests. **Results:** Integration of radiomic features significantly improved prognostic discrimination over clinical/RT plan data alone for both LLMs. For death prediction, pre-RT radiomics were the most predictive: GPT-4o achieved a peak AUC of 0.730 using intratumoral features, while Gemma-2-27b reached 0.736 using peritumoral features. For recurrence prediction, mid-RT peritumoral features yielded optimal performance (AUC = 0.703 for GPT-4o; AUC = 0.709 for Gemma-2-27b). Kaplan–Meier analyses confirmed statistically significant separation of risk groups: pre-RT intra- and peritumoral features for overall survival (for both GPT-4o and Gemma-2-27b, *p* < 0.05), and mid-RT peritumoral features for recurrence-free survival (*p* = 0.028 for GPT-4o; *p* = 0.017 for Gemma-2-27b). McNemar tests revealed no significant performance difference between the two LLMs when augmented with radiomics (all *p* > 0.05), indicating that the open-source model achieved comparable accuracy to its proprietary counterpart. Both models generated clinically coherent, patient-specific rationales explaining risk assignments, enhancing interpretability and clinical trust. **Conclusions:** This external validation demonstrates that pre-trained LLMs can serve as accurate, interpretable, and multimodal prognostic engines for LHC. Pre-RT radiomic features are critical for predicting mortality and metastasis, while mid-RT peritumoral features uniquely inform recurrence risk. The comparable performance of the open-source Gemma-2-27b-it model suggests a scalable, cost-effective, and privacy-preserving pathway for the integration of LLM-based tools into precision radiation oncology workflows to enhance risk stratification and therapeutic personalization.

## 1. Introduction

Laryngeal and hypopharyngeal cancers (LHC) are aggressive malignancies with poor outcomes [1,2]. Globally, they account for more than 268,000 new cases and approximately 138,000 deaths each year [3]. Standard treatments such as radiotherapy (RT) and chemotherapy often achieve only suboptimal disease control [4,5,6,7], underscoring the need for better tools to guide management and improve prognosis.

Treatment of LHC is particularly complex because clinicians must balance oncologic control with preservation of laryngeal function [8,9]. Despite current best practices, patient outcomes remain highly heterogeneous—ranging from durable control to early progression and treatment-related morbidity [10]. This variability highlights an urgent need for predictive tools that can anticipate individual treatment responses and support timely clinical decisions.

Radiomics has emerged as a promising, non-invasive approach for tumor characterization, extracting high-throughput imaging features that capture phenotypic and microenvironmental properties beyond visual inspection [11,12,13,14,15,16,17,18,19]. Computed tomography (CT), favored for its spatial resolution and accessibility, has shown particular utility. Prior studies suggest that CT-based radiomics can add prognostic value beyond TNM staging, improving prediction of pathologic features, treatment response, and survival [13,18]. However, radiomics models still lack standardization; performance can vary with modeling choices and small sample sizes, limiting generalizability and reproducibility [11]. Moreover, prognostic assessment after RT should also account for clinical status and treatment parameters—such as dose and plan details—which radiomics-only pipelines typically do not integrate [20,21,22].

The landscape of oncologic prognostication is rapidly evolving with the advent of generative AI. Recent 2024–2025 studies have highlighted the potential of Large Language Models (LLMs) to serve as powerful adjuncts in clinical oncology [23,24,25,26,27,28,29,30]. Beyond text-based tasks, the frontier is moving toward multimodal models that can integrate heterogeneous data streams—such as clinical reports, pathology findings, and imaging data [23,24,25,26,27,28,29,30]. This multimodal integration is critical, as it may address the known reliability and performance challenges of LLMs when operating on limited or unimodal information [23,24,25,26,27,28,29,30]. Our study is positioned within this emerging context, aiming to validate a multimodal framework that grounds LLM reasoning with quantitative imaging biomarkers.

LLMs represent a new avenue for oncology prognostication [31,32,33,34]. When supported by retrieval-augmented generation (RAG), LLMs can synthesize heterogeneous inputs, generate concise rationales, and reduce hallucinations [35,36]. This capacity addresses a common limitation of prior machine-learning and deep-learning approaches—their limited interpretability [37,38,39].

We therefore hypothesize that leveraging pre-trained LLMs to integrate clinical variables, RT plan information, and CT-derived radiomic features can provides significant prognostic value for patients with LHC treated with definitive (chemo)RT [40,41,42]. In this study, we aimed to externally validate the performance of two pre-trained LLMs for this task.

## 2. Materials and Methods

### 2.1. Patient Selection

We retrospectively reviewed patients treated at Linkou Chang Gung Memorial Hospital between January 2006 and December 2013. Eligible patients had newly diagnosed, pathologically confirmed squamous cell carcinoma of the larynx or hypopharynx without distant metastasis at presentation. All received definitive RT or concurrent chemoradiotherapy (CCRT). Non-contrast planning CT was performed before RT, and a mid-RT CT was obtained during RT to support adaptive planning. RT was standardized: all patients received an equivalent dose in 2-Gy fractions (EQD2) of ≥66.0 Gy, completed within 70 days. To minimize confounding, we excluded patients with any other primary malignancy within 3 years before index treatment.

All patients were stratified into high- and low-risk groups for three endpoints—death, recurrence, and distant metastasis. High-risk status was defined a priori as the occurrence of the respective event within 5 years. The study adhered to the Declaration of Helsinki and received approval from the Institutional Review Board (IRB 202300046B0); the requirement for written informed consent was waived owing to the retrospective design.

### 2.2. Image Acquisition and Preprocessing

Each patient underwent a non-contrast planning CT before treatment (pre-RT CT) and a repeat CT approximately 3 weeks after RT initiation (mid-RT CT) to assess interval change and enable plan adaptation. Primary tumors (the “intratumoral region”) were manually contoured on both scans by board-certified radiation oncologists (W-KY, C-YL, K-HF, B-SH, J-TC, and C-HL) following routine clinical protocols based on staging imaging. A peritumoral region was generated by uniformly expanding the intratumoral contour by 5 mm to capture potential microscopic extension.

Pre-RT and mid-RT CTs were rigidly co-registered to account for anatomical changes. Weekly nasofiberoscopy and physical examinations informed adaptive decisions, including recognition of tumor shrinkage (e.g., reduction in protrusions into air cavities or re-emergence of fat planes). Given heterogeneous, mosaic-like response patterns, initially involved soft-tissue infiltration was retained within targets to maintain oncologic safety. To focus on primary-tumor biology and organ preservation, lymph nodes were excluded from analysis.

To standardize image data, voxel spacing and slice thickness were harmonized via trilinear interpolation to 1.3 × 1.3 × 3.9 mm^3^. Within intra- and peritumoral masks, Hounsfield units were clipped to −200 to +400 to exclude air and bone, restricting analyses to soft tissues.

### 2.3. Radiomic Feature Extraction

Radiomic features were extracted using PyRadiomics (v3.1.0) in Python 3.11.9 on Ubuntu 20.04 [43]. To prioritize interpretability and clinical face-validity, we limited features to first-order statistics (18 items) and 3D shape descriptors (14 items), listed in Supplementary Table S1 [44]. More abstract texture features were intentionally excluded to avoid adding modeling complexity without transparent biological meaning. Features were computed from both intratumoral and peritumoral regions on pre-RT and mid-RT CTs, yielding four distinct sets: pre-RT intratumor, pre-RT peritumor, mid-RT intratumor, and mid-RT peritumor. Details regarding the development and validation of these radiomic sets have been reported in our previously published study [13].

A key design choice was to present these features directly to the LLMs rather than fitting separate classification/regression models; direct feature presentation preserves transparency of inputs and the LLMs’ reasoning.

### 2.4. LLM Selection

The selection of GPT-4o-2024-08-06 and Gemma-2-27b-it was strategically designed to compare the performance of two distinct archetypes of LLM deployment. GPT-4o was selected as the state-of-the-art, high-performance proprietary model, serving as a benchmark for maximal prognostic capability. In contrast, Gemma-2-27b-it was selected as a state-of-the-art, computationally efficient open-source model. This comparison is critical for assessing the clinical viability, cost-effectiveness, and scalability of deploying LLM-based prognostic tools, particularly for local, privacy-preserving implementation in resource-varied clinical settings.

We selected GPT-4o-2024-08-06 (OpenAI), which ranked at or near the top of the LMSYS Chatbot Arena leaderboard in August 2024. The LMSYS leaderboard aggregates large-scale, head-to-head human preference judgments across diverse tasks (conversation, problem solving, and contextual reasoning), providing a complementary perspective to static benchmarks that can be vulnerable to overfitting. A practical advantage of GPT-4o is its strong adherence to structured outputs (e.g., JSON), yielding reliable, instruction-following responses. We did not fine-tune GPT-4o because fine-tuning was not available for this model at the time of the study and our sample size was insufficient to justify training at this scale. Instead, we used a simple retrieval-augmented strategy (i.e., manually including all relevant patient data as context within the prompt), not an automated vector-retrieval pipeline [45,46,47]. To ensure reproducibility, all generative outputs for both models were obtained using deterministic (greedy) decoding by setting the sampling temperature parameter to 0 [45,46,47]. All results reflect a single, deterministic pass for each patient prompt [45,46,47].

To enhance reproducibility and reduce cost barriers, we also assessed Gemma-2-27b-it (Google), an open-source model that performs competitively among models runnable on consumer-grade hardware. We used the int4-quantized Unsloth build, which requires ~16 GB of RAM/VRAM with minimal degradation relative to full precision. Although Gemma-2-27b-it supports fine-tuning, for comparability we evaluated both models without task-specific fine-tuning. This zero-shot evaluation paradigm was necessitated by our modest sample size (N = 92), which is insufficient for robust fine-tuning, as well as the ‘black-box’ API nature of GPT-4o. This approach provides a crucial baseline for ‘out-of-the-box’ model performance on this novel and complex multimodal task. Because neither model was fine-tuned for regression, we restricted outputs to binary risk stratification (high vs. low risk) rather than continuous survival prediction.

### 2.5. Prompt Design for LLM Input

We designed prompt variants to assess how information richness affects prognostic performance. The baseline prompt contained only clinical variables and RT plan notes. Four enhanced prompts appended exactly one radiomic set—pre-RT intratumor, pre-RT peritumor, mid-RT intratumor, or mid-RT peritumor—to the same clinical/RT plan context. To limit cognitive load and avoid numeric brittleness in LLMs, we deliberately avoided cross-combining multiple radiomic sets within a single prompt. All 32 radiomic features were provided to the models as a single formatted list. Numeric precision was preserved as extracted. The order of features was fixed across all patients. To improve readability, the full prompt template is provided in Supplementary File S1.

### 2.6. Statistical Analysis

The primary endpoint was overall survival (OS), measured from the first day of RT to death or censoring (last follow-up). Secondary endpoints were recurrence-free survival (RFS) and distant metastasis-free survival (DMFS), each measured from RT start to the corresponding event or censoring. Median follow-up was estimated using the reverse Kaplan–Meier method.

Survival curves were generated with the Kaplan–Meier method and compared using log-rank tests; hazard ratios were estimated with Cox proportional hazards models (Wald tests). For each prompt condition, we calculated sensitivity, specificity, and the area under the receiver operating characteristic curve (AUC). Pairwise McNemar tests were used to compare paired binary classifications between models or prompt conditions. Statistical analyses were performed in Python 3.11.9 (Ubuntu 20.04) using statsmodels v0.14.2 and lifelines v0.29.0. Two-sided *p* values < 0.05 were considered statistically significant. We chose the McNemar test as it is the appropriate method for comparing paired, binary classifications (i.e., the ‘high’ vs. ‘low’ risk output) and directly assesses discordant predictions between the two models on the same patient [48]. This was chosen over other methods, such as DeLong’s test, which compares the overall AUC rather than the classification agreement [48]. Furthermore, given the exploratory, hypothesis-generating nature of this study on a modest (N = 92) cohort, a formal correction for multiple comparisons (e.g., Bonferroni) was not applied, as this would unduly inflate the risk of Type II error [49,50]; all *p*-values should therefore be interpreted as exploratory.

## 3. Results

### 3.1. Patients’ Characteristics

We presented the baseline and treatment characteristics of the patients in Table 1. A total of 92 patients were enrolled between 2006 and 2013. The median follow-up time was 7.4 years (95% CI: 6.8–8.0 years). The median age at diagnosis was 56 years (range, 34–80), and the vast majority were male (95.7%). Most patients presented with advanced disease, with 75.0% classified as T3–T4, 47.8% as N2–N3, and 70.6% staged as III–IVB. The hypopharynx was the predominant primary site (75.0%), followed by the larynx (25.0%). The median pre-RT tumor volume was 19.5 cm^3^ (range, 1.8–138.0), which decreased to 16.9 cm^3^ (range, 0.5–113.3) during RT.

Lifestyle risk factors were highly prevalent, including cigarette smoking (95.7%), betel quid chewing (71.7%), and alcohol consumption (54.3%). Comorbid medical conditions were documented in 65.2% of patients. Concurrent chemotherapy was administered in 72.8% of cases. The median overall treatment duration was 54 days (range, 47–68), with a median delivered equivalent dose in 2-Gy fractions (EQD2) of 72 Gy (range, 66–72). The median circulating lymphocyte count (CLC) prior to RT was 1979/mm^3^ (range, 645–5120), which declined to a nadir of 456/mm^3^ (range, 56–1456) during treatment. The proportions of patients classified as high risk for death, recurrence, and distant metastasis were 53.26% [high-risk (*n* = 49) and low-risk (*n* = 43)], 58.70% [high-risk (*n* = 54) and low-risk (*n* = 38)], and 54.35% [high-risk (*n* = 50) and low-risk (*n* = 42)], respectively.

### 3.2. Sensitivity, Specificity, and AUC

Table 2, Table 3 and Table 4 summarize sensitivity, specificity, and area under the ROC curve (AUC) for GPT-4o and Gemma-2-27b across endpoints and radiomic feature sets. For overall-survival prediction (Table 2), GPT-4o consistently showed higher specificity across all feature sets, with AUCs of 0.6465–0.7303. Gemma-2-27b achieved higher sensitivity when intra- or peritumoral features were included (0.8143–0.8551) but generally lower specificity (0.4953–0.6581), yielding slightly lower AUCs overall (0.6116–0.7362). For both models, inclusion of intra- and peritumoral features improved discrimination; peritumoral features produced the strongest OS performance (AUC 0.7303 for GPT-4o; 0.7362 for Gemma-2-27b).

For recurrence prediction (Table 3), both GPT-4o and Gemma-2-27b improved over the clinical/RT plan baseline when radiomic features were added. GPT-4o achieved its highest AUC with mid-RT CT peritumoral features (AUC = 0.7033). Although sensitivity was generally high, specificity remained modest across feature sets, peaking at 0.6000 with pre-RT CT intratumoral features. Gemma-2-27b likewise showed high sensitivity, with its best AUC using mid-RT CT peritumoral features (AUC = 0.7087); specificity was limited, with the highest value (0.5737) observed with both pre-RT CT intratumoral and peritumoral features. Notably, at baseline both models exhibited perfect sensitivity (1.0000) but poor specificity (0.3579 for GPT-4o; 0.2263 for Gemma-2-27b), resulting in low AUCs.

For distant metastasis prediction (Table 4), both models outperformed the clinical/RT plan baseline when radiomic features were added. Pre-RT CT intratumoral features provided the best discrimination (AUC 0.7195 for GPT-4o; 0.7276 for Gemma-2-27b). Although sensitivity improved, specificity remained modest across feature sets, indicating a tendency toward positive classification and yielding only moderate AUCs overall.

### 3.3. McNemar Test

**Figure 1:**
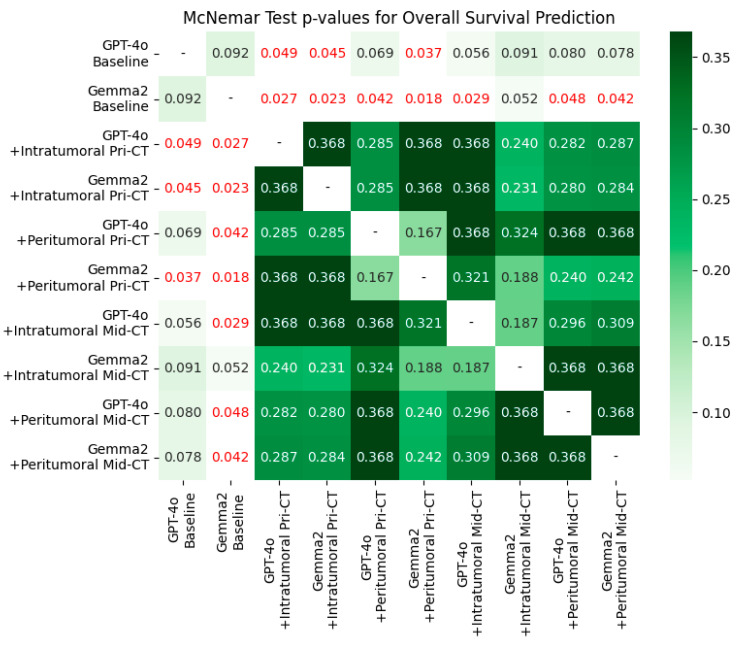
The McNemar test assessed the significance of differences between paired models for overall survival prediction using AUROC scores. Cells with *p* < 0.05 are highlighted in red.

**Figure 2:**
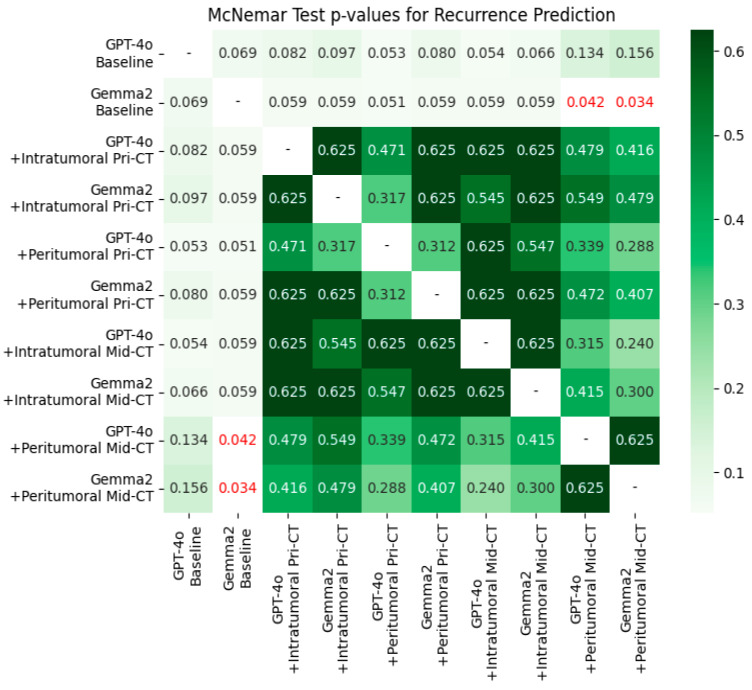
The McNemar test assessed the significance of differences between paired models for recurrence prediction using AUROC scores. Cells with *p* < 0.05 are highlighted in red.

**Figure 3:**
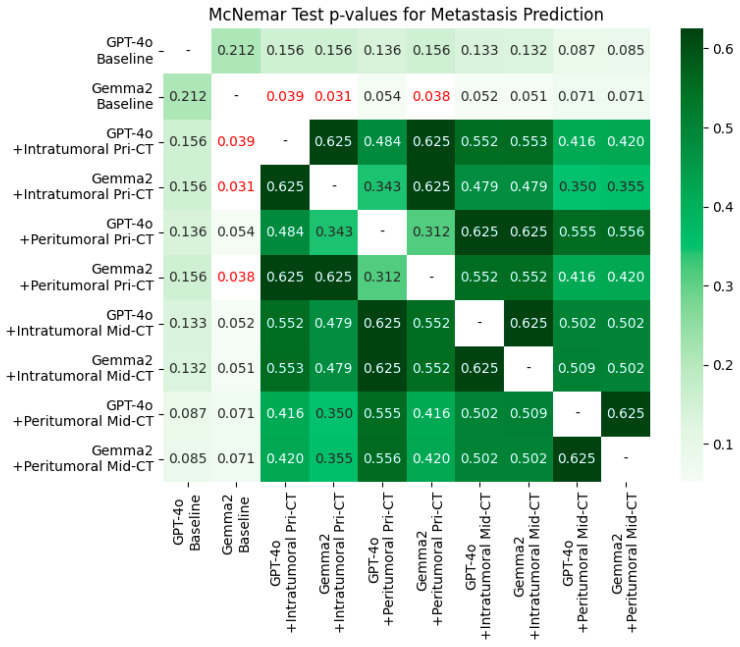
The McNemar test assessed the significance of differences between paired models for distant metastasis prediction using AUROC scores. Cells with *p* < 0.05 are highlighted in red.

Figure 1, Figure 2 and Figure 3 report McNemar tests comparing paired binary classifications between models. For OS (Figure 1), baseline performance did not differ significantly between GPT-4o and Gemma-2-27b (*p* = 0.092). After adding intratumoral or peritumoral CT radiomic features, each model improved significantly relative to its own baseline (all *p* < 0.05), with the largest gains observed for Gemma-2-27b. Across radiomics-enhanced conditions, no statistically significant differences were detected between the two models (all *p* > 0.05). Note: AUROC values are shown for context; McNemar tests assess discordant classifications rather than AUROC directly.

Figure 2 presents McNemar tests for recurrence prediction. At baseline, GPT-4o and Gemma-2-27b did not differ significantly (*p* = 0.069). After integrating radiomics (pre-RT or mid-RT; intratumoral or peritumoral), most comparisons with the corresponding baselines remained non-significant (all *p* > 0.05). The notable exception was Gemma-2-27b augmented with mid-RT CT peritumoral features, which showed a significant improvement over its baseline (*p* < 0.05). In contrast, adding intratumoral features did not yield significant gains relative to either model’s baseline.

Figure 3 displays a heatmap summarizing McNemar tests for distant metastasis prediction (paired classification comparisons); AUROC values are shown for context. Baseline performance did not differ significantly between GPT-4o and Gemma-2-27b (*p* = 0.212). Significant improvements (*p* < 0.05; cells highlighted in red) were observed primarily when models augmented with pre-RT CT intratumoral features were compared with the Gemma-2-27b baseline. In contrast, adding mid-RT CT radiomic features did not yield significant gains (all *p* > 0.05).

### 3.4. Kaplan–Meier Survival Analysis

Supplementary Figures S1 and S2 show Kaplan–Meier curves for OS, RFS, and DMFS under the GPT-4o and the Gemma-2-27b baseline models (clinical/RT plan only). Across all panels, the low-risk group exhibits higher survival probabilities, with the high-risk group declining more steeply. However, the models assigned relatively few patients to the low-risk group, creating substantial class imbalance. This imbalance limits statistical power for between-group comparisons.

**Figure 4:**
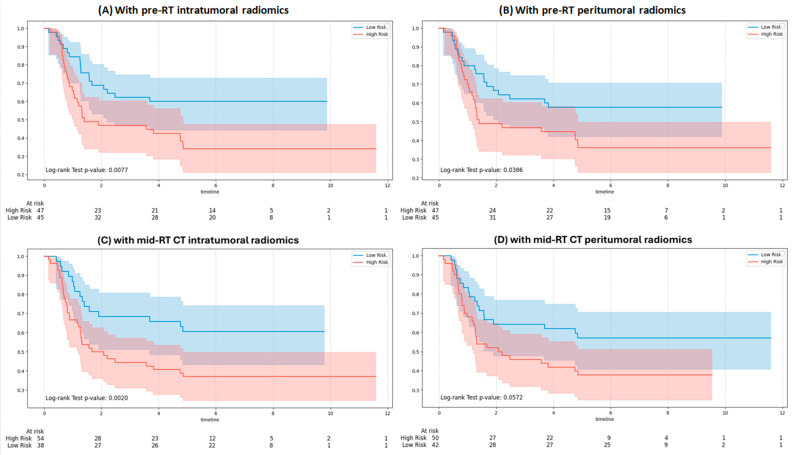
Kaplan–Meier survival curves for overall survival stratified by the GPT-4o model: (**A**) with pre-RT intratumoral features, (**B**) with pre-RT peritumoral features, (**C**) with mid-RT intratumoral features, and (**D**) with mid-RT peritumoral features.

Figure 4 shows KM curves for OS using GPT-4o integrating clinical and RT note variables with different radiomic inputs. Significant separation between high- and low-risk groups was observed with pre-RT intratumoral (*p* = 0.0077), pre-RT peritumoral (*p* = 0.0386), and mid-RT intratumoral features (*p* = 0.0020). In contrast, integration with mid-RT peritumoral features (Figure 4D) did not reach statistical significance (*p* = 0.0572).

**Figure 5:**
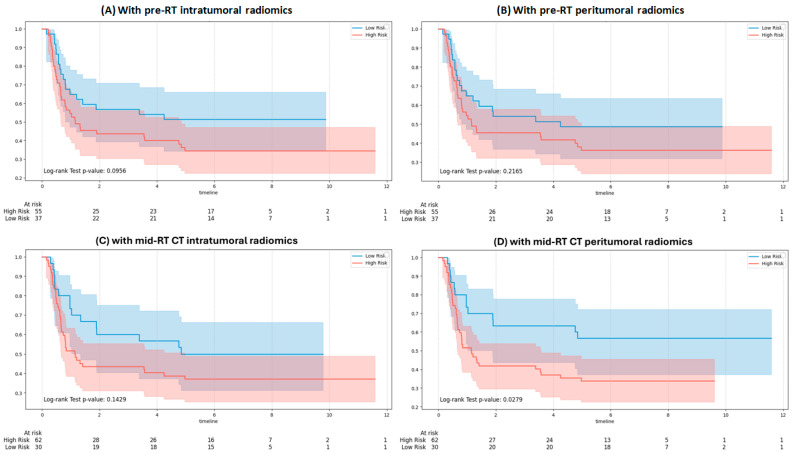
Kaplan–Meier survival curves for recurrence stratified by the GPT-4o model: (**A**) with pre-RT intratumoral features, (**B**) with pre-RT peritumoral features, (**C**) with mid-RT intratumoral features, and (**D**) with mid-RT peritumoral features.

Figure 5 shows KM curves for RFS using GPT-4o integrating clinical and RT note variables with pre-RT and mid-RT radiomic inputs. No significant separation was observed with pre-RT intratumoral (*p* = 0.0956), pre-RT peritumoral (*p* = 0.2165), or mid-RT intratumoral features (*p* = 0.1429). In contrast, integration with mid-RT peritumoral features (Figure 5D) achieved significant separation between risk groups (*p* = 0.0279).

**Figure 6:**
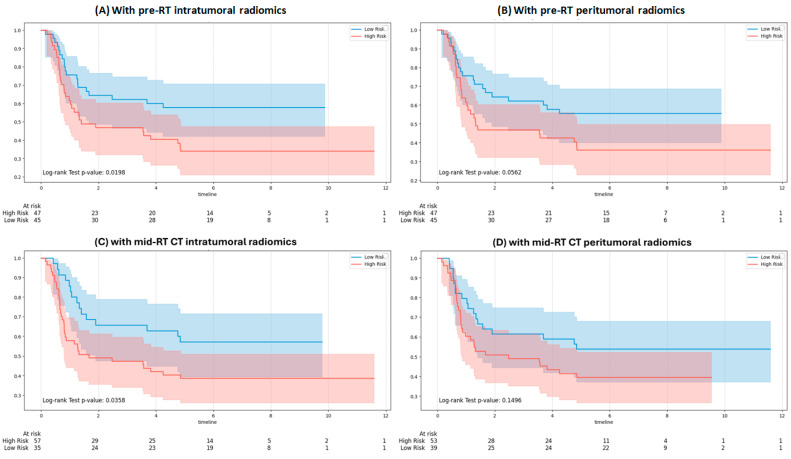
Kaplan–Meier survival curves for distant metastasis stratified by the GPT-4o model: (**A**) with pre-RT intratumoral features, (**B**) with pre-RT peritumoral features, (**C**) with mid-RT intratumoral features, and (**D**) with mid-RT peritumoral features.

Figure 6 presents KM curves for DMFS using GPT-4o integrating clinical and RT note variables with pre-RT and mid-RT radiomic inputs. Integration with pre-RT intratumoral features (Figure 6A) and mid-RT intratumoral features (Figure 6C) showed significant separation between risk groups (*p* = 0.0198 and *p* = 0.0358, respectively). In contrast, integration with pre-RT peritumoral features (Figure 6B) and mid-RT peritumoral features (Figure 6D) did not yield significant separation (*p* = 0.0562 and *p* = 0.1496, respectively).

**Figure 7:**
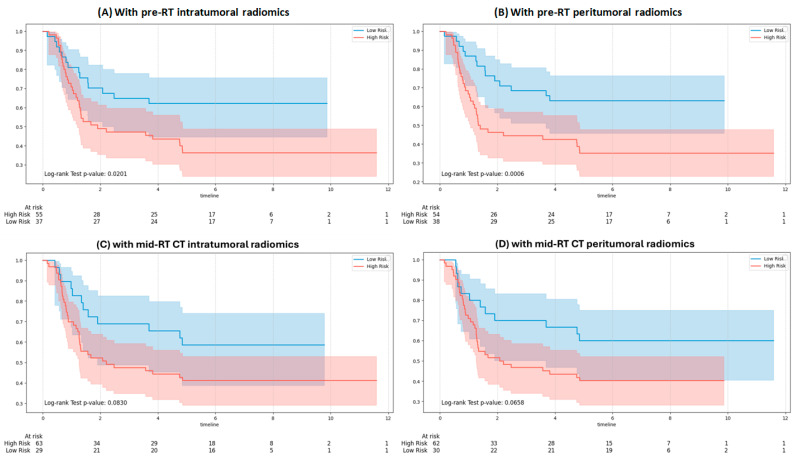
Kaplan–Meier survival curves for overall survival stratified by the Gemma-2-27b model: (**A**) with pre-RT intratumoral features, (**B**) with pre-RT peritumoral features, (**C**) with mid-RT intratumoral features, and (**D**) with mid-RT peritumoral features.

Figure 7 shows KM curves for OS using Gemma-2-27b integrating clinical and RT note variables with pre-RT and mid-RT radiomic inputs. Significant separation between risk groups was observed with pre-RT intratumoral (Figure 7A; *p* = 0.0201) and pre-RT peritumoral features (Figure 7B; *p* = 0.0006), indicating strong prognostic value. In contrast, mid-RT intratumoral (Figure 7C; *p* = 0.0830) and mid-RT peritumoral features (Figure 7D; *p* = 0.0658) did not reach statistical significance.

**Figure 8:**
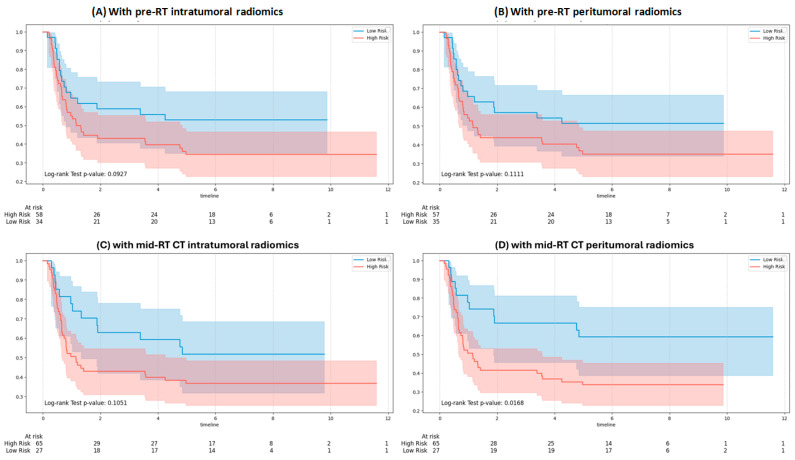
Kaplan–Meier survival curves for recurrence stratified by the Gemma-2-27b model: (**A**) with pre-RT intratumoral features, (**B**) with pre-RT peritumoral features, (**C**) with mid-RT intratumoral features, and (**D**) with mid-RT peritumoral features.

Figure 8 shows KM curves for RFS using Gemma-2-27b integrating clinical and RT note variables with pre-RT and mid-RT radiomic inputs. No significant separation was observed with pre-RT intratumoral (*p* = 0.0927), pre-RT peritumoral (*p* = 0.1111), or mid-RT intratumoral features (*p* = 0.1051). In contrast, mid-RT peritumoral features (Figure 8D) achieved significant separation (*p* = 0.0168), representing the strongest predictor of recurrence among the evaluated feature sets.

**Figure 9:**
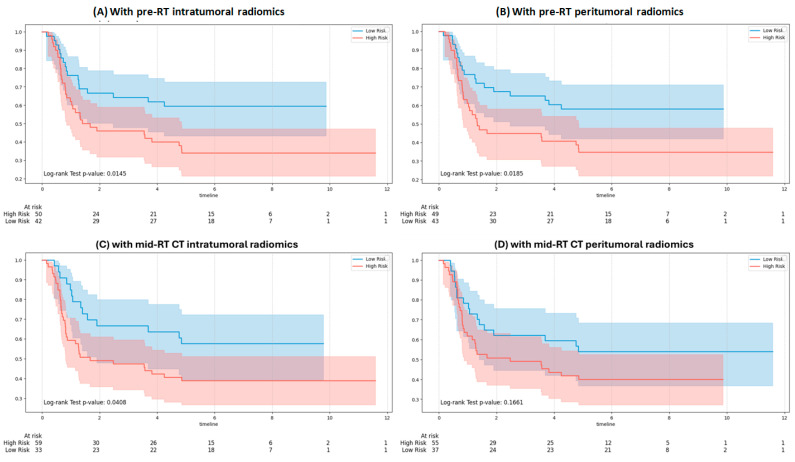
Kaplan–Meier survival curves for distant metastasis stratified by the Gemma-2-27b model: (**A**) with pre-RT intratumoral features, (**B**) with pre-RT peritumoral features, (**C**) with mid-RT intratumoral features, and (**D**) with mid-RT peritumoral features.

Figure 9 presents KM curves for DMFS using Gemma-2-27b integrating clinical and RT note variables with pre-RT and mid-RT radiomic inputs. Significant separation between risk groups was observed with pre-RT intratumoral (Figure 9A; *p* = 0.0145), pre-RT peritumoral (Figure 9B; *p* = 0.0185), and mid-RT intratumoral features (Figure 9C; *p* = 0.0408). In contrast, mid-RT peritumoral features (Figure 9D; *p* = 0.1661) did not reach statistical significance.

### 3.5. Qualitative Explanatory Reasoning

To illustrate this interpretability, we provide two representative examples of the model’s explanatory reasoning below.

(1) Combined with pre-RT intratumoral radiomic features to predict the outcome of death(−), recurrence(−), and metastasis(−):
  *“1. Tumor Staging and Burden: The absence of nodal disease (N0) significantly improves the prognosis. The reported initial tumor volume of 3.76 (units likely cubic centimeters) is relatively small for a T3 lesion.*
  *2. Clinical Risk Factors: At 50 years old, the patient is relatively younger typically with better overall performance status. Multiple phase-based dosing (e.g., higher-dose to the gross tumor volume, followed by lower-dose to subclinical regions) helps target both the primary tumor site and potential microscopic disease, improving cure rates.*
  *3. Pre-RT Intratumoral Radiomics Indicators: The reported Sphericity value (0.63405) suggests a relatively regular tumor shape, as extremely low sphericity often indicates more irregular or invasive growth. A more compact tumor can sometimes respond more uniformly to radiation. Elongation (0.839707) and moderate Flatness (0.424511) imply the tumor’s geometry is not extremely distorted; this may correlate with a more localized extension rather than multifocal spread. The Uniformity (0.253221) here is relatively moderate, suggesting the tumor’s intensity distribution might not be excessively chaotic.”*
(2) Combined with mid-RT peritumoral radiomic features to predict the outcome of death(+), recurrence(+), and metastasis(+):
  *“1. Tumor Staging and Burden: Advanced Stage T4a, N2 generally exhibit higher recurrence and metastasis rates. After three weeks of radiotherapy, the volume only reduced to 77.38, indicating limited response to treatment. Large tumors are often associated with hypoxic regions, leading to reduced radiosensitization and increased resistance to radiotherapy and chemotherapy drug penetration.*
  *2. Clinical Risk Factors: The 67 years of age with pre-existing comorbidities can impact treatment planning and tolerance. For instance, chronic disease-induced immunosuppression, treatment interruptions, or dose adjustments can lead to poor local control.*
  *3. Mid-RT Peritumoral Radiomics Indicators: Imaging reveals high “Surface Area” and large “Mesh Volume,” with relatively low “Sphericity” (0.195495), indicating irregular tumor shapes with significant surface undulations. These features often imply biologically complex tumor behavior with multiple invasion pathways. Texture features such as “Energy,”“Entropy,” and “Uniformity” demonstrate a chaotic density distribution, reflecting heterogeneity in tumor composition. This heterogeneity is often associated with higher malignancy and resistant cell populations.”*

## 4. Discussion

In this external validation study, we demonstrated that two pre-trained LLMs (GPT-4o and Gemma-2-27b) can serve as a multimodal prognostic framework for LHC. Our primary findings were consistent across both models: (1) The baseline models using only clinical/RT data had modest performance, (2) The integration of CT-derived radiomic features consistently and significantly improved prognostic discrimination, and (3) The optimal feature set depended on the clinical endpoint, with pre-RT features being most predictive for death and metastasis, while mid-RT features were most informative for recurrence.

Model operating characteristics suggested complementary strengths: Gemma-2-27b tended to be more sensitive, identifying a greater proportion of high-risk cases, whereas GPT-4o generally demonstrated higher specificity. Notably, despite slightly lower baseline AUCs, Gemma-2-27b realized larger gains with radiomic augmentation, indicating that a smaller open-source model can approach the performance of a larger proprietary model when supplied with informative, structured inputs.

A critical observation from our results (Table 2, Table 3 and Table 4) is the performance profile of the baseline models (using clinical/RT data only), which was characterized by exceptionally high sensitivity (often 1.0000) at the expense of modest specificity (e.g., 0.2930 for GPT-4o OS prediction). We interpret this finding in two essential contexts. First, from a clinical utility perspective, this high-sensitivity profile is a key characteristic of an effective ‘safety net’ or decision support tool. In a high-stakes field like oncologic prognostication, the consequence of a false negative (classifying a high-risk patient as low-risk) is far more severe than that of a false positive. A model that ‘errs on the side of caution’ by flagging all potential high-risk cases for human review is clinically acceptable and often desirable. Second, and most importantly, our study’s primary finding is that this trade-off is not static. The integration of radiomic features provided the necessary grounding to significantly improve this balance. For example, adding pre-RT intratumoral features (Table 2) improved GPT-4o’s specificity from 0.2930 to 0.7279, and Gemma-2’s specificity (with pre-RT peritumoral features) rose from 0.2233 to 0.6581. This demonstrates that multimodal data is crucial for anchoring the LLMs’ reasoning and mitigating the rate of false positives.

Conventional machine learning and deep learning approaches are frequently characterized by their opacity, which undermines interpretability and limits clinical adoption [11,12,13,14,15,16,17,18,19]. In contrast, large language models have the capacity to synthesize clinical, imaging, and treatment information while simultaneously articulating the rationale underlying their predictions. This capability directly addresses one of the principal barriers to the deployment of artificial intelligence–based prognostic tools in oncology.

A primary motivation for this study was to leverage the intrinsic explainability of generative models. The ‘chain-of-thought’ style reasoning provided in our examples (e.g., ‘Advanced Stage T4a, N2. indicating limited response’) offers a qualitative, human-readable rationale that is essential for building clinical trust and auditing a model’s logic. This contrasts with conventional ‘black box’ models where the reasoning pathway is opaque. However, this intrinsic ‘rationale’ is distinct from quantitative, post hoc eXplainable AI (XAI) methods such as LIME (Local Interpretable Model-agnostic Explanations) or SHAP (SHapley Additive exPlanations). These perturbation-based techniques provide feature-attribution scores, quantifying which specific inputs (e.g., ‘VoxelVolume’ or ‘T-stage’) most influenced a given prediction [51,52,53]. A comprehensive explainability framework would ideally include both. We have identified the application of these post hoc methods as a critical limitation and a key direction for future research.

Furthermore, our selection of generative, decoder-only transformer architectures (GPT-4o and Gemma-2-27b-it) was a deliberate choice predicated on our study’s dual objectives. While encoder-only models, such as Bidirectional Encoder Representations from Transformers (BERT), are highly effective for classification and natural language understanding (NLU) tasks, they are not designed for the generative component of our task: the production of a ‘chain-of-thought’ rationale [54]. Decoder-only models are the standard for such in-context generation and symbolic reasoning. Similarly, other generative architectures like Generative Adversarial Networks (GANs), while powerful for synthetic data generation or clinical trajectory forecasting, are less suited to our specific task of performing rationale-backed prognostication on structured multimodal inputs [55].

A key consideration is the integration of such a tool into existing oncology workflows. This framework is not intended to replace clinician judgment but to augment it as a Clinical Decision Support (CDS) tool. For instance, the system could be integrated into the Electronic Health Record (EHR) to automatically generate a prognostic report upon review of a new patient’s CT scan. This report, complete with the LLM’s ‘chain-of-thought’ reasoning, could then be presented to the multidisciplinary tumor board, providing a transparent ‘first opinion’ that clinicians can rapidly audit, critique, and verify, thereby facilitating more consistent and data-driven risk stratification.

Nevertheless, this study has several limitations that frame the interpretation of our findings and guide future research. First, and most critically, are the limitations imposed by our modest sample size (N = 92). This retrospective cohort size has two cascading implications. (a) It inherently limits the statistical power and generalizability of our findings. (b) It precluded the use of task-specific fine-tuning. As recent literature suggests, effective fine-tuning on specialized medical tasks often requires larger cohorts (e.g., N > 200–300) [56]. Our study was therefore restricted to a zero-shot paradigm. The modest baseline specificity we observed is an expected and known characteristic of zero-shot models when applied to complex, small-N medical tasks [57,58]. We therefore interpret our results as a baseline for zero-shot multimodal prognostication, not as the optimized performance achievable with fine-tuning [57,58].

Second, the scope of models evaluated was intentionally focused rather than comprehensive. As justified in our Methods, this study was a strategic comparison of two representative archetypes (proprietary SOTA vs. open-source local), not an exhaustive benchmark of all architectures (e.g., other VLMs, LCMs, MoEs). Such a broad comparison would require substantial computational and financial resources—particularly for MoE models, which have high VRAM requirements for inference—and was beyond the scope of this initial validation study [59,60]. Our study also lacks a direct comparison to traditional machine learning baselines (e.g., Logistic Regression or Random Forest) using the same multimodal inputs. Such a comparison is a crucial next step to formally benchmark the performance-versus-interpretability trade-off of this LLM-based reasoning approach. Third, our analysis of explainability was limited to the models’ intrinsic generative reasoning. We did not apply quantitative post hoc XAI methods like LIME or SHAP. The application of these methods was constrained by the ‘black-box’ API nature of GPT-4o and the methodological complexity of applying perturbation techniques to a zero-shot, multimodal prompt framework [51,52,53]. Future work should focus on applying SHAP/LIME to the locally deployed Gemma-2 model to quantify feature importance [51,52,53].

Fourth, our methodology, particularly with the proprietary GPT-4o, is vulnerable to model version drift. API-based models can be updated by the vendor without notice, meaning the model (and its results) evaluated in this study may differ from a future version, posing a significant challenge for long-term reproducibility. Fifth, the generative explanations, while a key feature, carry the risk of hallucinated reasoning. The LLM may generate a plausible-sounding rationale that is not factually grounded in the provided patient data, making clinician auditing essential. Addressing key implementation challenges, such as the risks of model version drift and ‘hallucinated reasoning’, will be critical. This further reinforces the necessity of a human-in-the-loop workflow and the value of stable, locally hosted open-source models for ensuring clinical safety and reproducibility.

Sixth, we did not report 95% confidence intervals (CIs) for our AUC estimates. Given our modest sample size (N = 92), standard CIs for AUCs are known to be unstable and imprecise [61]. We therefore omitted them to avoid implying a misleading level of statistical precision [61]. Seventh, our radiomic features are vulnerable to a lack of standardization in CT acquisition and reconstruction. While our single-institution cohort benefited from harmonized interpolation, it is well-established that inter-scanner variability in acquisition parameters (e.g., tube current, noise index) and reconstruction kernels can significantly impact the stability and reproducibility of radiomic features [62]. This non-biological variability poses a major challenge for the generalizability of any radiomic-based model, including ours, to new multi-center datasets. Finally, our analysis relied exclusively on CT imaging which—although accessible and cost-effective—lacks the functional and molecular information afforded by PET or MRI, thereby constraining comprehensive tumor characterization.

Future investigations should prioritize larger, prospective, multi-center cohorts to enhance external validity and mitigate selection bias. Standardizing CT acquisition and preprocessing across institutions will be critical for ensuring radiomic robustness and reproducibility [63]. Incorporating complementary imaging modalities such as PET and MRI may enrich CT-based analyses by providing functional and molecular insights. Future work should move beyond prompting with derived features and explore true multimodal (Vision-Language) models capable of directly processing imaging data (e.g., PET/CT pixels) alongside genomic and proteomic profiles, creating a more holistic and end-to-end prognostic framework [64,65]. Advanced architectures beyond generative transformers also warrant consideration. Neural operators, for instance, represent a distinct paradigm for modeling dynamic spatiotemporal processes and may provide computationally efficient alternatives [66,67]. In parallel, advances in adaptive RT should be leveraged, and longitudinal analyses of radiomic features across the treatment course should be pursued to elucidate response dynamics and long-term outcomes.

## 5. Conclusions

In this external validation study, we demonstrate that pre-trained large language models (LLMs), when serving as a multimodal integration engine for clinical, treatment, and radiomic data, provide significant prognostic value for patients with LHC. Our findings confirm that grounding these models with quantitative CT-derived radiomic features is essential, significantly improving risk discrimination over baseline clinical data alone. A key finding is the comparable prognostic accuracy of the open-source Gemma-2 model to the proprietary GPT-4o, suggesting a viable, scalable, and privacy-preserving pathway for deploying these advanced AI tools in real-world clinical decision-support workflows. Furthermore, we characterized the performance profile of these models in a zero-shot, small-data context, identifying a high-sensitivity/modest-specificity trade-off at baseline that is clinically relevant and significantly mitigated by multimodal inputs. While this study validates the intrinsic explainability of LLMs through their rationale-backed outputs, it also highlights the limitations of a zero-shot paradigm and frames the critical need for future work in fine-tuning on larger cohorts and applying quantitative XAI methods. These findings support the continued integration of LLM-based tools into precision radiation oncology to enhance risk stratification and therapeutic personalization.

## Figures and Tables

**Table 1:** Baseline clinical characteristics of patients with hypopharyngeal or laryngeal cancer treated with radiotherapy (*n* = 92).

Characteristics	*n* (%)
Accrual time	2006–2013
Median age, years	56 (34–80)
Male sex	88 (95.7)
Tumor stage	
T1–T2	23 (25.0)
T3–T4	69 (75.0)
Nodal stage	
N0–N1	48 (52.2)
N2–N3	44 (47.8)
Overall stage	
I	1 (1.1)
II	5 (5.5)
III	21 (22.8)
IVA	52 (56.5)
IVB	13 (14.1)
Primary tumor site	
Hypopharynx	69 (75.0)
Larynx	23 (25.0)
Median tumor volume (pre-RT), cm^3^	19.5 (1.8–138.0)
Median tumor volume (mid-RT), cm^3^	16.9 (0.5–113.3)
Cigarette smoking	88 (95.7)
Betel quid chewing	66 (71.7)
Alcohol drinking	50 (54.3)
Presence of medical comorbidities	60 (65.2)
Chemotherapy	67 (72.8)
Median treatment duration, days	54 (47–68)
Median EQD2, Gy	72 (66–72)
Median CLC (pre-RT), per mm^3^	1979 (645–5120)
Median CLC (nadir during RT), per mm^3^	456 (56–1456)

**Table 2:** The sensitivity, specificity, and AUC of risk classification across different LLM pipelines for overall survival prediction. A 5-year cut-off was applied to balance the sample sizes of high-risk (*n* = 49) and low-risk (*n* = 43) groups.

OS	Baseline	Pre-RT Intra-T	Pre-RT Peri-T	Mid-RT Intra-T	Mid-RT Peri-T
GPT-4o-2024-08-06					
Sensitivity	1.0000	0.7327	0.7122	0.7939	0.7327
Specificity	0.2930	0.7279	0.7047	0.6349	0.6581
AUC	0.6465	0.7303	0.7084	0.7144	0.6954
Gemma-2-27b-it					
Sensitivity	1.0000	0.8143	0.8143	0.8551	0.8551
Specificity	0.2233	0.6349	0.6581	0.4953	0.5186
AUC	0.6116	0.7246	0.7362	0.6752	0.6869

**Table 3:** The sensitivity, specificity, and AUC of risk classification across different LLM pipelines for recurrence prediction. A 5-year cut-off was applied to balance the sample sizes of high-risk (*n* = 54) and low-risk (*n* = 38) groups.

Recurrence	Baseline	Pre-RT Intra-T	Pre-RT Peri-T	Mid-RT Intra-T	Mid-RT Peri-T
GPT-4o-2024-08-06					
Sensitivity	1.0000	0.7667	0.7481	0.8222	0.8593
Specificity	0.3579	0.6000	0.5737	0.4947	0.5474
AUC	0.6789	0.6833	0.6609	0.6585	0.7033
Gemma-2-27b-it					
Sensitivity	1.0000	0.8037	0.7852	0.8593	0.8963
Specificity	0.2263	0.5737	0.5737	0.4684	0.5211
AUC	0.6132	0.6887	0.6794	0.6638	0.7087

**Table 4:** The sensitivity, specificity, and AUC of risk classification across different LLM pipelines for metastasis prediction. A 5-year cut-off was applied to balance the sample sizes of high-risk (*n* = 50) and low-risk (*n* = 42) groups.

Metastasis	Baseline	Pre-RT Intra-T	Pre-RT Peri-T	Mid-RT Intra-T	Mid-RT Peri-T
GPT-4o-2024-08-06					
Sensitivity	0.8800	0.7201	0.7000	0.8000	0.7392
Specificity	0.5286	0.7188	0.6952	0.5762	0.6012
AUC	0.7043	0.7195	0.6976	0.6881	0.6700
Gemma-2-27b-it					
Sensitivity	0.9800	0.7600	0.7400	0.8200	0.7600
Specificity	0.2714	0.6952	0.6952	0.5524	0.5762
AUC	0.6257	0.7276	0.7176	0.6862	0.6681

## Data Availability

The data that support the findings of this study are available from the corresponding author upon reasonable request. The data are not publicly available due to the sensitive nature of the patient data (which includes detailed clinical, treatment, and imaging information) and the restrictions imposed by our Institutional Review Board.

## Supplementary Materials

The following supporting information can be downloaded at: https://www.mdpi.com/article/10.3390/bioengineering12121345/s1, Figure S1. Kaplan-Meier survival curves for OS (left), RFS (middle), and DMFS (right), categorized by GPT-4o baseline model into high-risk (red) and low-risk (blue) groups. Figure S2. Kaplan-Meier survival curves for overall survival (left), recurrence-free survival (middle), and metastasis-free survival (right), categorized by Gemma-2-27b baseline model into high-risk (red) and low-risk (blue) groups. Table S1. Radiomic Features. Supplementary File S1. The full prompt template.

## References

[1] Amar A., de Almeida J.R., Kanda J.L., de Paula S.M.T., Lessa M.M. (2013). Epidemiological assessment and therapeutic response in hypopharyngeal cancer. Braz. J. Otorhinolaryngol..

[2] Luo X., Yu F., Xu C., Deng Z., Zeng Y., Zhao X., Zeng X. (2022). Evaluation of the prevalence of metachronous second primary malignancies in hypopharyngeal carcinoma and their effect on outcomes. Cancer Med..

[3] Bray F., Laversanne M., Sung H., de Martel C., Ferlay J., Brooks F., Mery L. (2024). Global cancer statistics 2022: GLOBOCAN estimates of incidence and mortality worldwide for 36 cancers in 185 countries. CA Cancer J. Clin..

[4] Baumann R., Linge A., Zips D. (2016). Targeting hypoxia to overcome radiation resistance in head & neck cancers: Real challenge or clinical fairytale?. Expert Rev. Anticancer Ther..

[5] Huang G., Pan S.T. (2020). ROS-Mediated Therapeutic Strategy in Chemo-/Radiotherapy of Head and Neck Cancer. Oxidative Med. Cell. Longev..

[6] Liu C., Wang P., Xiao R., Jiang W., Zhao X., Liu S., Li T., Zhang W., Li D., Chen D. (2020). Homologous recombination enhances radioresistance in hypopharyngeal cancer cell line by targeting DNA damage response. Oral Oncol..

[7] Zhong J.T., Zhou S.H. (2017). Warburg effect, hexokinase-II, and radioresistance of laryngeal carcinoma. Oncotarget.

[8] Calvas O.I.J., Dedivitis R.A., Pfuetzenreiter R., Filho W.J.M., Santos C.R.B. (2017). Oncological results of surgical treatment versus organ-function preservation in larynx and hypopharynx cancer. Rev. Assoc. Med. Bras..

[9] Qian W., Chen M., Feng G., Zhu X., Shi J., Zhang W., Han J., Wang W. (2014). Multi-modality management for loco-regionally advanced laryngeal and hypopharyngeal cancer: Balancing the benefit of efficacy and functional preservation. Med. Oncol..

[10] Newman J.R., Johnson J., Hornig J.D. (2015). Survival trends in hypopharyngeal cancer: A population-based review. Laryngoscope.

[11] Chiesa-Estomba C.M., Lopez-Flores R., Rivas M., Rivera-Lomeli B., Lopez-Perez V., Larranaga-Eguren Z., Azaola-Gutierrez M.P., Fernandez-Moyano A., Aliyeva T. (2023). Radiomics in Hypopharyngeal Cancer Management: A State-of-the-Art Review. Biomedicines.

[12] Liao K.Y., Chiu C.C., Chiang W.C., Chiou Y.R., Zhang G., Yang S.N., Huang T.C. (2019). Radiomics features analysis of PET images in oropharyngeal and hypopharyngeal cancer. Medicine.

[13] Lin C.H., Yan J.L., Yap W.K., Kang C.J., Chang Y.C., Tsai T.Y., Chang K.P., Liao C.T., Hsu C.L., Chou W.C. (2023). Prognostic value of interim CT-based peritumoral and intratumoral radiomics in laryngeal and hypopharyngeal cancer patients undergoing definitive radiotherapy. Radiother. Oncol..

[14] Lin Y.C., Lin G., Pandey S., Yeh C.H., Wang J.J., Lin C.Y., Ho T.Y., Ko S.F., Ng S.H. (2023). Fully automated segmentation and radiomics feature extraction of hypopharyngeal cancer on MRI using deep learning. Eur. Radiol..

[15] Mo X., Wei W., Xu X., Zhang T., Huang S. (2020). Prognostic value of the radiomics-based model in progression-free survival of hypopharyngeal cancer treated with chemoradiation. Eur. Radiol..

[16] Siow T.Y., Yeh C.H., Lin G., Lin C.Y., Wang H.M., Liao C.T., Toh C.H., Chan S.C., Lin C.P., Ng S.H. (2022). MRI Radiomics for Predicting Survival in Patients with Locally Advanced Hypopharyngeal Cancer Treated with Concurrent Chemoradiotherapy. Cancers.

[17] Su C.W., Tsan D.L., Hsu C.L., Tseng C.Y., Lin C.H., Fan K.H., Huang Y.C., Lin Y.C., Cheng Y.F., Wang W.H. (2022). Delta-volume radiomics of induction chemotherapy to predict outcome of subsequent chemoradiotherapy for locally advanced hypopharyngeal cancer. Tumori J..

[18] Wang Y., Lei D. (2022). Research progress in CT-based radiomics constructing hypopharyngeal cancer and multisystem tumor prediction model. Lin Chuang Er Bi Yan Hou Tou Jing Wai Ke Za Zhi.

[19] Wu T.C., Wu W.T., Lin C.Y., Tseng H.C., Yang Y.W., Jheng Y.C., Chang T.H., Chien Y.C., Fan K.H., Chen Y.C. (2024). Radiomics analysis for the prediction of locoregional recurrence of locally advanced oropharyngeal cancer and hypopharyngeal cancer. Eur. Arch. Otorhinolaryngol..

[20] Machtay M., Lee J.H., Moughan J., Trotti A., Garden A.S., Weber R.S., Harris J. (2012). Hypopharyngeal dose is associated with severe late toxicity in locally advanced head-and-neck cancer: An RTOG analysis. Int. J. Radiat. Oncol. Biol. Phys..

[21] Miah A.B., Bhide S.A., Newbold K.L., Clark C.H., Webster G., Gothard L., Dearnaley D.P., Rowbottom C.G., A’hern R.P., Sohaib S.A. (2012). Dose-escalated intensity-modulated radiotherapy is feasible and may improve locoregional control and laryngeal preservation in laryngo-hypopharyngeal cancers. Int. J. Radiat. Oncol. Biol. Phys..

[22] Yom S.S., Torres-Saavedra E., Caudell J.J., Waldron J.N., Spencer S., Saba N.F., Sturgis E.M., Axelrod R.S., Teknos T.N., Trotti A. (2021). Reduced-Dose Radiation Therapy for HPV-Associated Oropharyngeal Carcinoma (NRG Oncology HN002). J. Clin. Oncol..

[23] Bhuyan S.S., Islam N. (2025). Generative Artificial Intelligence Use in Healthcare: Opportunities for Clinical Excellence and Administrative Efficiency. J. Med. Syst..

[24] Fahim Y.A., Khan H., Zafarmand M., Ezzat K.M., Ahmed F., Moustafa A.M., Zaki M.M., Sayed S., Eltobgy M.A., Albalawi H.S. (2025). Artificial intelligence in healthcare and medicine: Clinical applications, therapeutic advances, and future perspectives. Eur. J. Med. Res..

[25] Hao Y., He H., Gu M., Chen F., Zhang J., Li Y., Wang H., Hu Z. (2025). Large language model integrations in cancer decision-making: A systematic review and meta-analysis. npj Digit. Med..

[26] Ah-Thiane L., Heudel P.-E., Campone M., Robert M., Brillaud-Meflah P., Rousseau C., Le Blanc-Onfroy M., Tomaszewski F., Supiot S., Perennec T. (2025). Large Language Models as Decision-Making Tools in Oncology: Comparing Artificial Intelligence Suggestions and Expert Recommendations. JCO Clin. Cancer Inform..

[27] Chen D., Li J., Lin C., Zhang Y., Yu J. (2025). Large language models in oncology: A review. BMJ Oncol..

[28] Gong E.J., Li W., Li S., Xu B., Jiang Z., Chen D., Chen W., Wang Y. (2024). The Potential Clinical Utility of the Customized Large Language Model in Gastroenterology: A Pilot Study. Bioengineering.

[29] Yu Y., Zhu J., Sun H., Xu J., Sun J. (2024). Using Large Language Models to Retrieve Critical Data from Clinical Processes and Business Rules. Bioengineering.

[30] Geantă M., Bădescu D., Chirca N., Nechita O.C., Radu C.G., Rascu Ș., Rădăvoi D., Sima C., Toma C., Jinga V. (2024). The Emerging Role of Large Language Models in Improving Prostate Cancer Literacy. Bioengineering.

[31] Aubreville M., Ganz J., Ammeling J., Rosbach E., Gehrke T., Scherzad A., Hackenberg S., Goncalves M. (2025). Prediction of tumor board procedural recommendations using large language models. Eur. Arch. Otorhinolaryngol..

[32] Chao P.J., Chang C.H., Wu J.J., Liu Y.H., Shiau J., Shih H.H., Lin G.Z., Lee S.H., Lee T.F. (2024). Improving Prediction of Complications Post-Proton Therapy in Lung Cancer Using Large Language Models and Meta-Analysis. Cancer Control.

[33] Jiang L.Y., Chen Y., Lunsford L.D., Chen Y., Zhang Y.T., Dligach D., Moons K.G.M., Hsieh C., Natarajan K., Savova G.K. (2023). Health system-scale language models are all-purpose prediction engines. Nature.

[34] Sun D., Yu R., Zhao S., Zheng W., Wang Z., Zhao Z. (2024). Outcome Prediction Using Multi-Modal Information: Integrating Large Language Model-Extracted Clinical Information and Image Analysis. Cancers.

[35] Unlu O., Duman Z.B., Kucuk A. (2024). Retrieval Augmented Generation Enabled Generative Pre-Trained Transformer 4 (GPT-4) Performance for Clinical Trial Screening. medRxiv.

[36] Zakka C., Shad R., Chaurasia A., Dalal A.R., Kim J.L., Moor M., Fong R., Phillips C., Alexander K., Ashley E. (2024). Almanac—Retrieval-Augmented Language Models for Clinical Medicine. NEJM AI.

[37] Petch J., Di S., Nelson W. (2022). Opening the Black Box: The Promise and Limitations of Explainable Machine Learning in Cardiology. Can. J. Cardiol..

[38] Rudin C. (2019). Stop Explaining Black Box Machine Learning Models for High Stakes Decisions and Use Interpretable Models Instead. Nat. Mach. Intell..

[39] Watson D.S., Krutzinna J., Bruce I.N., Griffiths C.E., McInnes I.B., Barnes M.R., Floridi L. (2019). Clinical applications of machine learning algorithms: Beyond the black box. BMJ.

[40] Cabral S., Restrepo D., Kanjee Z., Wilson P., Crowe B., Abdulnour R.E., Rodman A. (2024). Clinical Reasoning of a Generative Artificial Intelligence Model Compared With Physicians. JAMA Intern. Med..

[41] Savage T., Nayak A., Gallo R., Rangan E., Chen J.H. (2024). Diagnostic reasoning prompts reveal the potential for large language model interpretability in medicine. npj Digit. Med..

[42] Singhal K., Azizi S., Tu T., Mahdavi S.S., Wei J., Chung H.W., Scales N., Tanwani A., Cole-Lewis H., Pfohl S. (2023). Large language models encode clinical knowledge. Nature.

[43] Peng J., Lu F., Huang J., Zhang J., Gong W., Hu Y., Wang J. (2022). Development and validation of a pyradiomics signature to predict initial treatment response and prognosis during transarterial chemoembolization in hepatocellular carcinoma. Front. Oncol..

[44] Zwanenburg A., Vallières S., Sechopoulos I.O., Aerts H. (2020). The Image Biomarker Standardization Initiative: Standardized Quantitative Radiomics for High-Throughput Image-based Phenotyping. Radiology.

[45] Myers S., Miller T.A., Gao Y., Churpek M.M., Mayampurath A., Dligach D., Afshar M. (2025). Lessons learned on information retrieval in electronic health records: A comparison of embedding models and pooling strategies. J. Am. Med. Inform. Assoc..

[46] Tripathi S., Alkhulaifat D., Lyo S., Sukumaran R., Li B., Acharya V., McBeth R., Cook T.S. (2025). A Hitchhiker’s Guide to Good Prompting Practices for Large Language Models in Radiology. J. Am. Coll. Radiol..

[47] Aftab W., Apostolou Z., Bouazoune K., Straub T. (2024). Optimizing biomedical information retrieval with a keyword frequency-driven prompt enhancement strategy. BMC Bioinform..

[48] Fagerland M.W., Lydersen S., Laake P. (2013). The McNemar test for binary matched-pairs data: Mid-p and asymptotic are better than exact conditional. BMC Med. Res. Methodol..

[49] Greenland S. (2021). Analysis goals, error-cost sensitivity, and analysis hacking: Essential considerations in hypothesis testing and multiple comparisons. Paediatr. Perinat. Epidemiol..

[50] Armstrong R.A. (2014). When to use the Bonferroni correction. Ophthalmic Physiol. Opt..

[51] Hassan S.U., Mahmood A., Hussain A., Khan W.A. (2025). Local interpretable model-agnostic explanation approach for medical imaging analysis: A systematic literature review. Comput. Biol. Med..

[52] Haue A.D., Hjaltelin J.X., Holm P.C., Placido D., Brunak S.R. (2024). Artificial intelligence-aided data mining of medical records for cancer detection and screening. Lancet Oncol..

[53] He J., Wang X., Zhu P., Wang X., Zhang Y., Zhao J., Sun W., Hu K., He W., Xie J. (2025). Identification and validation of an explainable early-stage chronic kidney disease prediction model: A multicenter retrospective study. EClinicalMedicine.

[54] McCoy R.T., Yao S., Friedman D., Hardy M.D., Griffiths T.L. (2024). Embers of autoregression show how large language models are shaped by the problem they are trained to solve. Proc. Natl. Acad. Sci. USA.

[55] Shankar V., Wu W., Zhang W., Wang J., Cheng C., Zhang R., Lee Y., Li K., Deng T., Sun S. (2023). Clinical-GAN: Trajectory Forecasting of Clinical Events using Transformer and Generative Adversarial Networks. Artif. Intell. Med..

[56] Riley R.D., Ensor J., Sutton A.J., Moons K.G.M., Collins G.S., van Smeden M., Debray T.P.A., Snell K.I.E. (2019). Minimum sample size for developing a multivariable prediction model: Part I—Continuous outcomes. Stat. Med..

[57] Redekop E., Razzaghi T., Chen Y., Rizzolo D., Li X., Dligach D., Moons K.G.M., Hsieh C., Natarajan K., Savova G.K. (2025). Zero-shot medical event prediction using a generative pretrained transformer on electronic health records. J. Am. Med. Inform. Assoc..

[58] Naliyatthaliyazchayil P., Albalushi M., Alkindi N., Damanhori N., Al-Adawi S., Ambusaidi M., Al-Tobi M. (2025). Evaluating the Reasoning Capabilities of Large Language Models for Medical Coding and Hospital Readmission Risk Stratification: Zero-Shot Prompting Approach. J. Med. Internet Res..

[59] Han X., Zhang Z., Xu H., Huang J., Xu B., Jiang T., Lu S., Li S., Xu Y. (2025). ViMoE: An Empirical Study of Designing Vision Mixture-of-Experts. IEEE Trans. Image Process..

[60] Xu P., Wu Y., Jiang T., Chen Y., Yu P., Chen Y., Zheng Q., Wei W. (2024). LVLM-EHub: A Comprehensive Evaluation Benchmark for Large Vision-Language Models. IEEE Trans. Pattern Anal. Mach. Intell..

[61] Feng D., Zhang W., Ma H., Zuo T. (2017). A comparison of confidence/credible interval methods for the area under the ROC curve for continuous diagnostic tests with small sample size. Stat. Methods Med. Res..

[62] Reiazi R., Mehdi S., Ghadirzadeh M. (2021). The impact of the variation of imaging parameters on the robustness of Computed Tomography radiomic features: A review. Comput. Biol. Med..

[63] Levi R., Kelleher D., Fode K., Varmark C., Hansen M., Therkildsen J., Støttrup C., Sørensen L., Hansen R. (2024). A reference framework for standardization and harmonization of CT radiomics features on cadaveric sample. Sci. Rep..

[64] Xiang J., Zhao Y., Zhang R., Chen T., He Y., Li J., Li W., Li S., Zhang W. (2025). A vision-language foundation model for precision oncology. Nature.

[65] Nguyen H.H., Blaschko M.B., Saarakkala S., Tiulpin A. (2024). Clinically-Inspired Multi-Agent Transformers for Disease Trajectory Forecasting From Multimodal Data. IEEE Trans. Med. Imaging.

[66] Kaczmarek W., Magdziarz Ibrahim-El-Nur J., Łoś M., Roleder T. (2025). Reconstructing cerebral hemodynamics from sparse data using Neural Operator Transformers. Comput. Biol. Med..

[67] Sakovich N., Dmitry Aksenov D., Pleshakova E., Gataullin S. (2025). A neural operator using dynamic mode decomposition analysis to approximate partial differential equations. AIMS Math..

