# Supplementary material for: An External Validation Study on Two Pre-Trained Large Language Models for Multimodal Prognostication in Laryngeal and Hypopharyngeal Cancer: Integrating Clinical, Treatment, and Radiomic Data to Predict Survival Outcomes with Interpretable Reasoning
Source: Bioengineering (Basel). 2025 Dec 10;12(12):1345. doi: 10.3390/bioengineering12121345 (PMC12729448; doi:10.3390/bioengineering12121345)
Supplement: Supplementary file 1 [file bioengineering-12-01345-s001.zip › Supplementary Figures S1.pdf]

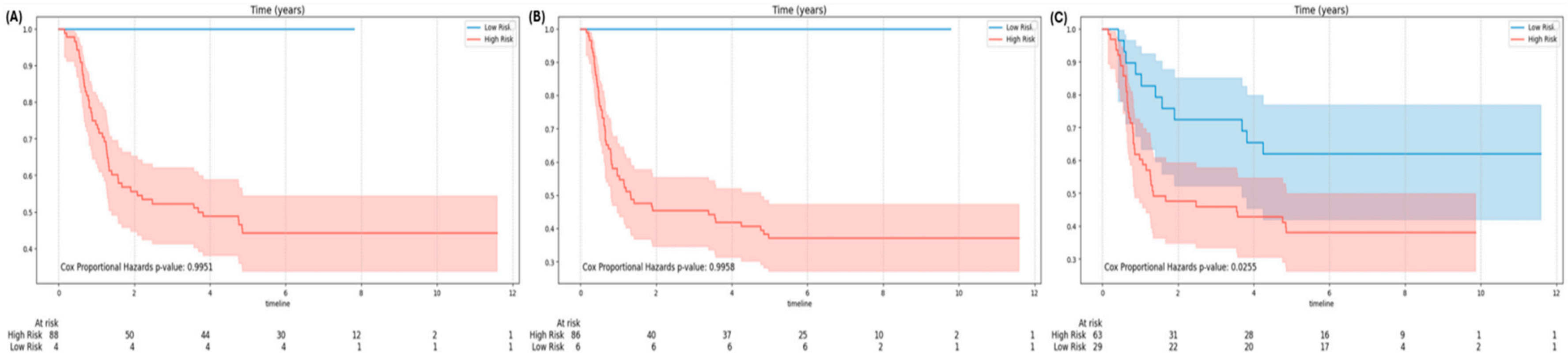

Figure S1. Kaplan-Meier survival curves for OS (left), RFS (middle), and DMFS (right), categorized by GPT-4o baseline model into high-risk (red) and low-risk (blue) groups.

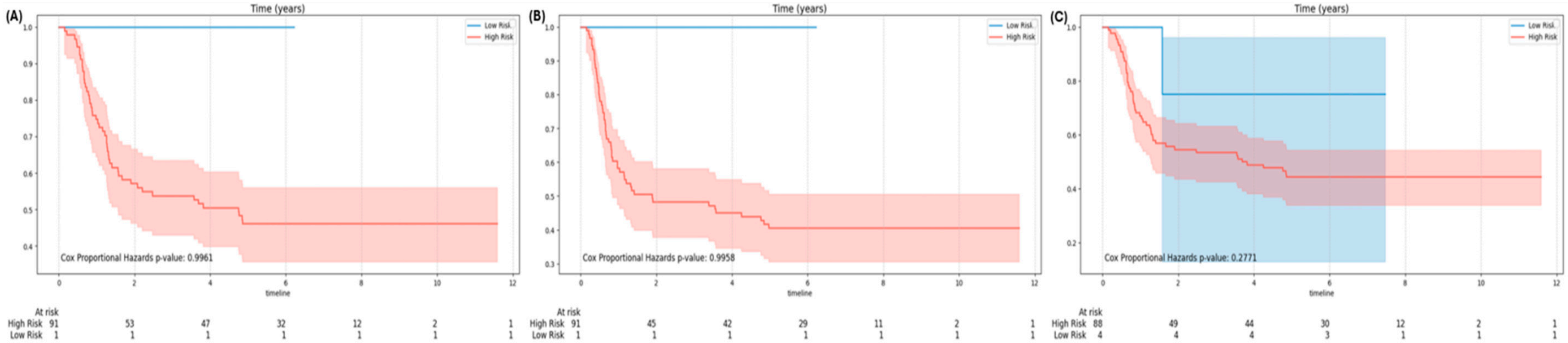

Figure S2. Kaplan-Meier survival curves for overall survival (left), recurrence-free survival (middle), and metastasis-free survival (right), categorized by Gemma-2-27b baseline model into high-risk (red) and low-risk (blue) groups.
