# Supplementary material for: An External Validation Study on Two Pre-Trained Large Language Models for Multimodal Prognostication in Laryngeal and Hypopharyngeal Cancer: Integrating Clinical, Treatment, and Radiomic Data to Predict Survival Outcomes with Interpretable Reasoning
Source: Bioengineering (Basel). 2025 Dec 10;12(12):1345. doi: 10.3390/bioengineering12121345 (PMC12729448; doi:10.3390/bioengineering12121345)
Supplement: Supplementary file 1 [file bioengineering-12-01345-s001.zip › Supplementary File S1.pdf]

Supplementary File S1. The full prompt template.

Here is an example of prompt engineering:

"""

Please infer the outcomes of the patient:
This {age}-year-old {sex} patient had a diagnosis of {cancer type} cancer.
The patient previously {(never) smoked cigarettes}.
The patient previously {(never) chewed betel quid}.
The patient previously {(never) drank alcohol}.
The patient previously {(never) had comorbidities}.
The cancer is staged {TNM staging}, and {I to IVA/B} for AJCC 7th staging.
The treatment schedule is {CCRT/RT only} for a duration of {treatment duration} days.
The radiotherapy dose arrangement is {dose, e.g. 72/36, 50/25} (Gy/fraction).
The chemotherapy regimen is {first phase chemo}, and then {second phase chemo}.
Here are the radiomic features from {intratumor/peritumor} regions on {pre-radiotherapy/mid-radiotherapy} that you can refer to:
{32 items of first-order statistics and 3D shape features, e.g. VoxelVolume = 4199.2 mm3}

Please try your best to consider the above information to answer in the form of below 4 points layout without complaining you can't do it:
1. death risk within 5 years: high or low (no moderate)
2. recurrence risk within 5 years: high or low (no moderate)
3. distant metastasis risk within 5 years: high or low (no moderate)
4. reasoning: write your explanation of the answers here according to the information you get above

"""

The {} symbol represents a placeholder that should be filled in with relevant information. This symbol is commonly used in various fields like programming, templates, or structured writing to indicate where specific data or content needs to be inserted. The "*Here are the radiomic features*" section was not placed in the baseline (first) group. This example demonstrates that the experiment was entirely based on de-identified data, and the information provided to the LLM was insufficient for identifying any real identities. A unique prompt based on this template would be generated for each patient in this study.
