# Supplementary material for: An External Validation Study on Two Pre-Trained Large Language Models for Multimodal Prognostication in Laryngeal and Hypopharyngeal Cancer: Integrating Clinical, Treatment, and Radiomic Data to Predict Survival Outcomes with Interpretable Reasoning
Source: Bioengineering (Basel). 2025 Dec 10;12(12):1345. doi: 10.3390/bioengineering12121345 (PMC12729448; doi:10.3390/bioengineering12121345)
Supplement: Supplementary file 1 [file bioengineering-12-01345-s001.zip › Supplementary Table S1.pdf]

Table S1: Radiomic Features

**First-Order Statistical Features (18)**	**Three-Dimensional Shape Descriptors (14)**
Mean	Volume
Median	Surface Area
Minimum	Surface Volume Ratio
Maximum	Compactness 1
Range	Compactness 2
Variance	Sphericity
Standard Deviation	Spherical Disproportion
Skewness	Maximum 3D Diameter
Kurtosis	Maximum 2D Diameter (Slice)
Energy	Maximum 2D Diameter (Column)
Entropy	Maximum 2D Diameter (Row)
Uniformity	Major Axis Length
Mean Absolute Deviation	Minor Axis Length
Robust Mean Absolute Deviation	Least Axis Length
Root Mean Squared	Elongation
10th Percentile	Flatness
90th Percentile	
Interquartile Range	
